# Development and validation of machine learning models and nomograms for predicting the surgical difficulty of laparoscopic resection in rectal cancer

**DOI:** 10.1186/s12957-024-03389-3

**Published:** 2024-04-25

**Authors:** Xiangyong Li, Zeyang Zhou, Bing Zhu, Yong Wu, Chungen Xing

**Affiliations:** 1 Department of Gastrointestinal Surgery, The Second Affiliated Hospital of Soochow University, Suzhou, Jiangsu province China; 2 Department of Anesthesiology, Dongtai People’s Hospital, Yancheng, Jiangsu Province China

**Keywords:** Rectal cancer, Surgical difficulty, Machine learning, Nomogram, Prediction model

## Abstract

**Background:**

The objective of this study is to develop and validate a machine learning (ML) prediction model for the assessment of laparoscopic total mesorectal excision (LaTME) surgery difficulty, as well as to identify independent risk factors that influence surgical difficulty. Establishing a nomogram aims to assist clinical practitioners in formulating more effective surgical plans before the procedure.

**Methods:**

This study included 186 patients with rectal cancer who underwent LaTME from January 2018 to December 2020. They were divided into a training cohort (*n* = 131) versus a validation cohort (*n* = 55). The difficulty of LaTME was defined based on Escal’s et al. scoring criteria with modifications. We utilized Lasso regression to screen the preoperative clinical characteristic variables and intraoperative information most relevant to surgical difficulty for the development and validation of four ML models: logistic regression (LR), support vector machine (SVM), random forest (RF), and decision tree (DT). The performance of the model was assessed based on the area under the receiver operating characteristic curve(AUC), sensitivity, specificity, and accuracy. Logistic regression-based column-line plots were created to visualize the predictive model. Consistency statistics (C-statistic) and calibration curves were used to discriminate and calibrate the nomogram, respectively.

**Results:**

In the validation cohort, all four ML models demonstrate good performance: SVM AUC = 0.987, RF AUC = 0.953, LR AUC = 0.950, and DT AUC = 0.904. To enhance visual evaluation, a logistic regression-based nomogram has been established. Predictive factors included in the nomogram are body mass index (BMI), distance between the tumor to the dentate line ≤ 10 cm, radiodensity of visceral adipose tissue (VAT), area of subcutaneous adipose tissue (SAT), tumor diameter >3 cm, and comorbid hypertension.

**Conclusion:**

In this study, four ML models based on intraoperative and preoperative risk factors and a nomogram based on logistic regression may be of help to surgeons in evaluating the surgical difficulty before operation and adopting appropriate responses and surgical protocols.

**Supplementary Information:**

The online version contains supplementary material available at 10.1186/s12957-024-03389-3.

## Background

Colorectal cancer is the third most common cancer in the world and the second leading cause of cancer deaths, with rectal cancer (RC) accounting for one-third of the total [1, 2]. Despite the availability of various therapeutic options, surgical resection is still the primary treatment for early- to mid-stage and even partially advanced rectal cancer (RC) [3]. Laparoscopic total mesorectal excision (LaTME) is recognized as the surgical technique of choice for the treatment of rectal cancer, with a pathologic safety profile and overall survival rate no less than that of open surgery [4]. However, due to the complexity and high level of difficulty of the procedure, intraoperative conversion to open surgery and complications are relatively common. Although these conditions do not indicate surgical failure, they significantly affect the prognosis [4, 5]. Therefore, preoperative surgical evaluation is particularly important.

Multiple studies have been conducted to confirm the consequences of obesity on surgical difficulties, with most focusing on BMI [6–8]. While BMI is an estimation of generalized obesity, it does not accurately reflect the relationship with surgical difficulties. Therefore, the assessment of abdominal fat is deemed more significant compared to BMI [9]. The use of computed tomography (CT) to measure the area and mean radiodensity of abdominal visceral adipose tissue and subcutaneous adipose tissue has been validated in the majority of studies [10, 11], ensuring its reliability. The inclusion of the area and radiodensity of visceral adipose tissue and subcutaneous adipose tissue in this study allows for a more comprehensive assessment of obesity concerning surgical difficulty.

Machine learning (ML), a burgeoning form of artificial intelligence (AI), is increasingly being applied in healthcare data analysis. ML excels in connecting multiple variables and accurately predicting outcomes. Consequently, several ML prediction models are currently being adopted for disease diagnosis, prognosis prediction, and clinical decision-making [12, 13], and the objective of this study was to utilize machine learning with nomograms to establish a preoperative prediction model for the surgical difficulty of laparoscopic rectal cancer surgery, to identify preoperative independent predictors of surgical difficulty that consequence the difficulty of the surgery, and consequently, to help medical surgeons to develop personalized surgical options for their patients preoperatively.

## Materials and methods

### Patients

The Ethics Committee of the Second Affiliated Hospital of Soochow University approved our retrospective study. It conforms to the 1964 Helsinki Declaration of the World Medical Association and its subsequent revisions. This study included 186 patients who underwent laparoscopic total mesorectal excision (LaTME) from January 2018 to December 2020 in the Department of Gastrointestinal Surgery of the Second Affiliated Hospital of Soochow University.

The inclusion criteria were:

(1)Preoperative pathological examination that confirmed the diagnosis of rectal cancer; (2) complete computed tomography (CT) scan and clinical data within two weeks before surgery; (3) preoperative plan for laparoscopic total mesorectal excision (LaTME).

The exclusion criteria were :

(1) Emergency surgery; (2) open surgery; (3) preoperative adjuvant treatment such as radiotherapy or chemotherapy; (4) clinical stage 4 or huge tumor that was inoperable. Clinicopathologic parameters were retrospectively collected from the medical record database. The Clavien-Dindo classification [14] was utilized to classify postoperative short-term complications.

### Data collection

For patients included in the study, the following parameters are retrospectively collected from our hospital’s electronic medical record system: (1) Basic patient characteristics: age, gender, BMI, American Society of Anesthesiologists (ASA) score, and comorbidities (hypertension, diabetes). (2) Laboratory results: albumin, hemoglobin, triglycerides, total cholesterol, C-reactive protein, and Systemic Inflammatory Grade (SIG) [15]within two weeks before surgery. (3) Intraoperative data: surgical time and the need for conversion. (4) Postoperative data: pathological results, hospitalization time, and postoperative complications classified according to the Clavien-Dindo classification.

### CT abdominal adipose tissue measurements

Visceral adipose tissue (VAT) and subcutaneous adipose tissue (SAT) parameters are calculated and averaged using Slice-O-Matic software (version 5.0; TomoVision) at the level of the fourth and fifth lumbar vertebral interspaces by selecting two consecutive CT cross-Sect. (5 mm). Cross-sectional areas are delineated based on anatomical knowledge and tissue-specific Hounsfield unit (HU) ranges of -150 to -50 HU for VAT and − 190 to -30 HU for SAT [16]. For two CT scans of the same patient, regions of interest are outlined separately by two individuals trained in software usage and then averaged (Supplementary Fig. S1). If substantial differences exist between the two outlines, a third person reviews and verifies the measurements.

### Surgical difficulty criteria

Based on the criteria established by Escal et al. [17] and in combination with the research by Y. Seki et al. [9], which demonstrates that patients with a visceral fat area/body surface area (VFA/BSA) ≥ 85 cm2/m2 present greater surgical challenges, refinements have been made to formulate the scoring criteria. The scoring system encompasses five factors related to surgical difficulty, each factor being assigned a weighting based on clinical experience. Scores range from 0 to 10 points and are divided into two categories: a cumulative score below 3 points indicates non-difficult surgery, while a score of 3 points or higher signifies surgical difficulty (Table 1). The five variables associated with surgical difficulty show significant differences between the surgical-difficulty group and the non-surgical difficulty group (*P* < 0.05) (Supplementary Table S1).

**Table 1:** Surgical difficulty grading

	Points
Duration of surgery >300 min	3
Conversion to open procedure	3
Postoperative hospital stay >14 days	2
VAT/BAS>85 cm2/m2	1
Morbidity (grade II and III)	1

*(VFA /BSA)* visceral fat area/body surface area

### Establishment of machine learning models

**Fig. 1:**
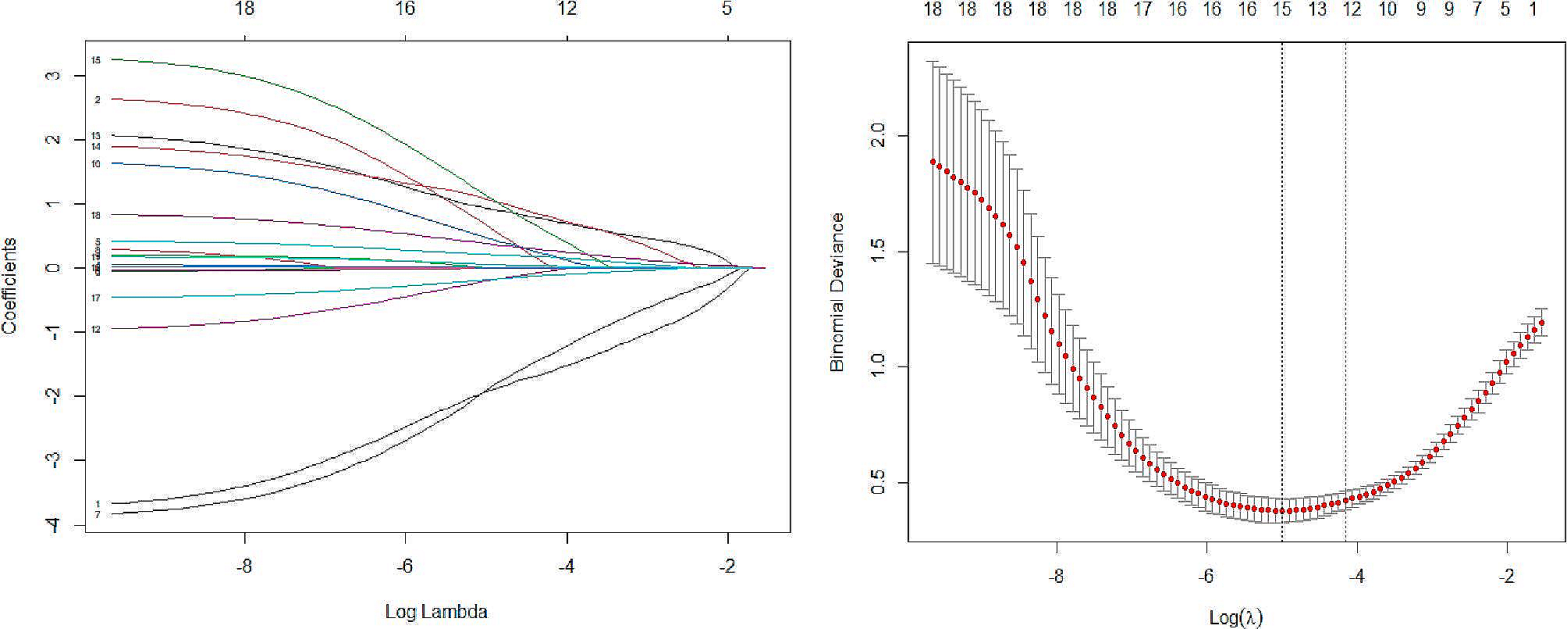
(**A**) LASSO coefficient profile for the 21 variables. (**B**) Selection of the best penalty coefficient λ in the LASSO model, using 10-fold cross-validation based on the minimum criterion. The dashed line on the left represents lambda. min and the dashed line on the right represents lambda.1se

We utilized R software (version 4.3.1) to set a fixed random seed of grouping and randomly divided all patients into two groups according to 7:3, training cohort (*n* = 131) and validation cohort (*n* = 55). To simplify the machine learning model and enhance its generalization capability, 10 cross-validated LASSO regressions are employed to reduce variable dimensionality. The filtering criterion is based on the lambda. min variable, with the most optimal model fit observed at lambda.min = 0.007424. Fifteen out of the twenty-one variables with the highest predictive power for surgical difficulties are selected (Fig. 1). It has also been demonstrated that age [18], and ASA [19] have an consequence on the performance of the procedure, and therefore they were included as model predictor variables.

In this study, a total of four machine learning models were selected to predict the degree of surgical difficulty: support vector machine (SVC), random forest (RF), logistic regression (LR), and decision tree (DT). In the training cohort, we individually parameterized all models to tune to prevent overfitting or underfitting, and the same hyperparameters were used in the validation cohort to assess the predictive ability of the models. In the training cohort, we adjusted the parameters of all models to prevent overfitting or underfitting and used the same hyperparameters in the validation cohort to evaluate the predictive ability of the models.

### Statistical analysis

Statistical analyses are conducted using the statistical R software (version 4.3.1) package. Continuous data that adhere to a normal distribution are presented as mean and standard deviation (SD), while non-normally distributed continuous data are displayed as median and interquartile range (IQR). Categorical data are represented as frequency and percentage (%). T-tests are utilized for the comparison of continuous variables, and χ2 tests are employed for the comparison of categorical variables. Predictive nomograms are developed. The variables selected for inclusion in the nomogram are determined through the backward stepwise method of Akaike’s information criterion, and factors with a multifactor logistic regression *P* < 0.05 are included. Calibration curves are created to evaluate the calibration of the nomogram. Furthermore, Harrell’s C-index is calculated, and bootstrap validation of the nomogram is performed (using 1000 bootstrap weight samples) to compute the C-index of relative calibration. Decision curve analysis (DCA) is also conducted. Statistical significance is defined as *P* < 0.05.

## Results

### Patient characteristics

A total of 186 patients were included in the final analysis, including 112 males (60.22%) and 74 females (39.78%). The median age was 66 years, the median BMI was 23.35 kg/m2, 76 had comorbid hypertension, and 21 had diabetes mellitus.101 had tumors > 3 cm in diameter, 88 had tumors to the dentate line ≥ 10 cm, the median visceral adipose tissue radiodensity was − 94.08 U, the median subcutaneous adipose tissue radiodensity was − 97.34 U, and the median subcutaneous adipose tissue area median was 127.55 cm2. 147 had preoperative serum albumin ≥ 35 g/L. There were no significant differences in clinical characteristics and CT parameters between the training and the validation cohorts (*P* > 0.05) (Table 2).

**Table 2:** Clinical characteristics and CT parameters in the training cohort and validation cohort

Variable	Total (n = 186)	Training cohort (n = 131)	Validation cohort (n = 55)	P
**Baseline characteristics of the patients**				
Age(mean [SD], year)	66 ± 11	66.57 ± 11	64 ± 10	0.322
BMI (median [IQR], kg/m²)	23.35 (21.63–25.48)	23.44 (21.50–25.59)	23.18 (21.97–25.20)	0.847
Gender, n (%)				0.772
Female	74 (39.78)	53 (40.46)	21 (38.18)	
Male	112 (60.22)	78 (59.54)	34 (61.82)	
ASA, n (%)				0.726
1	22 (11.83)	17 (12.98)	5 (9.09)	
2	114 (61.29)	80 (61.07)	34 (61.82)	
3	50 (26.88)	34 (25.95)	16 (29.09)	
Hypertension, n (%)				0.863
No	110 (59.14)	78 (59.54)	32 (58.18)	
Yes	76 (40.86)	53 (40.46)	23 (41.82)	
Diabetes, n (%)				0.157
No	165 (88.71)	119 (90.84)	46 (83.64)	
Yes	21 (11.29)	12 (9.16)	9 (16.36)	
**CT measurement parameters**				
SAT area(mean [SD], cm2)	127.55 ± 55.04	128.57 ± 59.40	125.13 ± 43.29	0.699
SAT Radiodensity(mean [SD], U)	-97.34 ± 9.03	-96.95 ± 8.73	-98.27 ± 7.10	0.367
VAT Radiodensity( mean [SD], U)	-94.08 ± 8.36	-94.18 ± 8.57	-93.83 ± 7.89	0.793
Distance between tumor and the dentate line, n (%)				0.053
< 10cm	98 (52.69)	63 (48.09)	35 (63.64)	
≥ 10 cm	88 (47.31)	68 (51.91)	20 (36.36)	
Tumor diameter, n (%)				0.355
≤ 3 cm	85 (45.7)	57 (43.51)	28 (50.91)	
>3 cm	101 (54.3)	74 (56.49)	27 (49.09)	
**Pathological stage**				
Pathological T stage, n (%)				0.982
T1	132 (70.97)	93 (70.99)	39 (70.91)	
T2	43 (23.12)	30 (22.90)	13 (23.64)	
T3	11 (5.91)	8 (6.11)	3 (5.45)	
Pathological N stage, n (%)				0.195
N0	112 (60.22)	75 (57.25)	37 (67.27)	
N1	39 (20.97)	27 (20.61)	12 (21.82)	
N2	35 (18.82)	29 (22.14)	6 (10.91)	
Nerve invasion, n (%)				0.396
No	146 (78.49)	105 (80.15)	41 (74.55)	
Yes	40 (21.51)	26 (19.85)	14 (25.45)	
Vascular tumor emboli, n (%)				0.661
No	152 (81.72)	106 (80.92)	46 (83.64)	
Yes	34 (18.28)	25 (19.08)	9 (16.36)	
**Blood Laboratory indicators**				
Preoperative total cholesterol (median [IQR])	4.63 (4.24–5.20)	4.59 (4.17–5.11)	4.65 (4.30–5.23)	0.381
Preoperative triglyceride(median [IQR])	1.18 (0.95–1.45)	1.23 (1.01–1.46)	1.09 (0.71–1.38)	0.021
Preoperative C reactive protein count(median [IQR])	5.20 (4.70–5.70)	5.10 (4.70–5.70)	5.50 (4.90–5.70)	0.058
Preoperative serum albumin, n (%)				0.854
<35 g/L	39 (20.97)	27 (20.61)	12 (21.82)	
≥ 35 g/L	147 (79.03)	104 (79.39)	43 (78.18)	
Preoperative hemoglobin (mean [SD])	129.78 ± 17.67	128.90 ± 16.78	131.87 ± 19.63	0.296
SIG, n (%)				0.22
0	121 (65.05)	87 (66.41)	34 (61.82)	
1	46 (24.73)	31 (23.66)	15 (27.27)	
2	13 (6.99)	7 (5.34)	6 (10.91)	
3	6 (3.23)	6 (4.58)	0 (0.00)	

*BMI* body mass index; *ASA* American Society of Anesthesiologists; *SAT* subcutaneous fatty tissue; *VAT* visceral adipose tissue; *SIG* Systemic Inflammatory Grade

### Construction of machine learning models

**Fig. 2:**
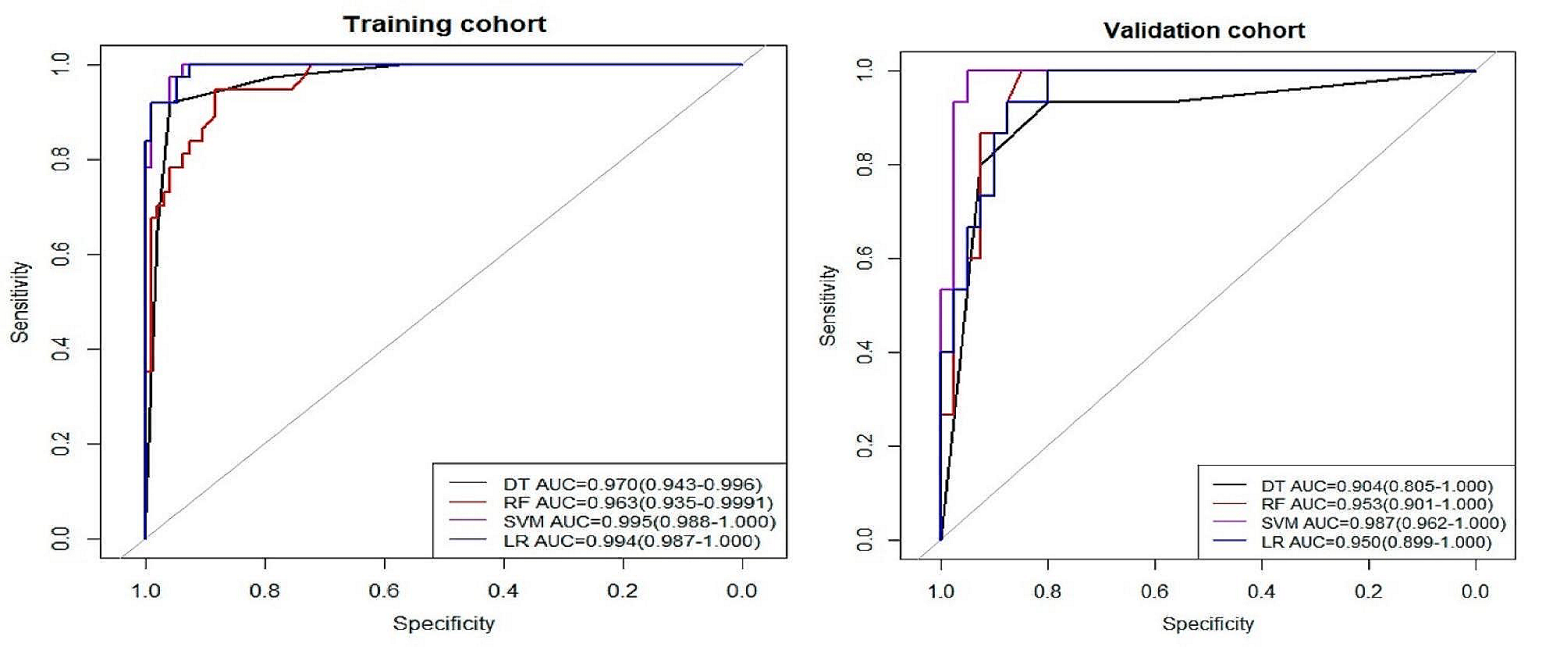
Evaluation of the receiver operating characteristic curve (ROC) performance for four machine learning models based on the area under the receiver operating characteristic curve (AUC) in training (**A**) and validation (**B**) cohorts

Four machine learning models - Support Vector Machine (SVC), Random Forest (RF), Logistic Regression (LR), and Decision Tree (DT) - were selected for this study. The optimal hyperparameters were calculated by repeating the cross-validation five times considering the accuracy and AUC. Subsequently, the optimal hyperparameters were derived by manual tuning. We also obtained the following parameters: sensitivity, specificity, precision, recall, and F1 (Table 3). Using AUC as the evaluation criterion, the optimal performance in the training cohort: SVM AUC = 0.995 (0.988-1.000), the other models are LR AUC = 0.994 (0.987-1.000), DT AUC = 0.970 (0.943–0.996) and RF AUC = 0.963 (0.935–0.999). The best performance in the validation cohort: SVM AUC = 0.987 (0.962-1.000) and the other models are RF AUC = 0.953 (0.901-1.000), LR AUC = 0.950 (0.889-1.000) and DT AUC = 0.904 (0.805-1.000)( Fig. 2). The De-Long test is employed to compare the predictive efficacy of the four machine learning models, the results indicated no significant differences among the models (*P* > 0.05), all of which demonstrated superior predictive performance (Supplementary Table S2).

**Table 3:** Performance of four machine learning models in training and validation cohorts

					Training Cohort					Validation Cohort				
Model	AUC	Accuracy	Sensitivity	Specificity		Recall	F1 Score	AUC	Accuracy		Sensitivity	Specificity	Recall	F1 Score
SVM	0.995	0.954	0.936	1		0.936	0.96	0.987	0.855		0.8	1	0.8	0.889
DT	0.97	0.9466	0.919	0.957		0.919	0.907	0.904	0.836		0.933	0.8	0.933	0.757
RF	0.963	0.9	0.946	0.883		0.946	0.843	0.953	0.873		0.867	0.875	0.867	0.788
LR	0.994	0.969	0.971	0.967		0.919	0.944	0.95	0.855		0.733	0.9	0.733	0.733

### Results of univariate and multivariate logistic regression analysis

After univariate and multivariate logistic regression, it was identified that BMI (OR:1.52, 95% CI: 1.10–2.11), SAT area (OR:1.02, 95% CI: 1.01–1.04), VAT radiodensity (OR:1.34, 95% CI: 1.16–1.56), the distance between the tumor and the dentate line < 10 cm (OR:0.03, 95% CI: 0.01–0.21), tumor diameter > 3 cm (OR:0.14, 95% CI: 0.03–0.82), and comorbid hypertension (OR:0.19, 95% CI: 0.04–0.83) were independent risk factors for surgical difficulties( Table 4).

**Table 4:** Univariate and multivariate logistic regression analysis between the training and validation cohorts

Variables	Univariate		Multivariate	
	OR (95%CI)	*P*	OR (95%CI)	*P*
Age	1.02 (0.98–1.05)	0.307		
Gender				
Female	Reference			
Male	1.37 (0.62–3.01)	0.437		
BMI	1.12 (1.01–1.26)	0.047	1.52 (1.10–2.11)	0.011
ASA				
2	Reference			
3	0.34 (0.12–0.97)	0.044	0.64 (0.10–4.27)	0.642
1	0.82 (0.26–2.56)	0.730	1.86 (0.28–12.36)	0.52
Hypertension				
Yes	Reference			
No	0.14 (0.06–0.32)	< 0.001	0.20 (0.05–0.87)	0.032
Preoperative hemoglobin	1.00 (0.98–1.02)	0.886		
Preoperative serum albumin				
≥ 35 g/L	Reference			
<35 g/L	5.75 (2.33–14.21)	< 0.001	5.10 (0.91–28.60)	0.064
Preoperative total cholesterol	0.87 (0.54–1.40)	0.565		
Preoperative triglyceride	1.61 (0.89–2.88)	0.112		
Preoperative C -reactive protein count	1.00 (0.99–1.02)	0.759		
SAT area	1.01 (1.01–1.01)	0.045	1.02 (1.01–1.04)	0.013
SAT Radiodensity	0.96 (0.91–1.01)	0.098		
VAT Radiodensity	1.12 (1.06–1.18)	< 0.001	1.34 (1.16–1.56)	**< 0.001**
Distance between tumor and the dentate line				
<10 cm	Reference			
≥ 10 cm	0.13 (0.05–0.32)	< 0.001	0.03 (0.01–0.21)	**< 0.001**
SIG				
3	Reference			
0	2.50 (0.28–22.40)	0.413		
1	1.20 (0.12–12.27)	0.878		
2	0.83 (0.04–16.99)	0.906		
Tumor diameter				
>3 cm	Reference			
≤ 3 cm	0.10 (0.03–0.30)	< 0.001	0.14 (0.03–0.82)	0.029
Diabetes				
No	Reference			
Yes	1.30 (0.37–4.62)	0.682		

*BMI* body mass index; *ASA* American Society of Anesthesiologists; *SAT* subcutaneous fatty tissue; *VAT* visceral adipose tissue; *SIG* Systemic Inflammatory Grade

### Development and validation of nomogram

**Fig. 3:**
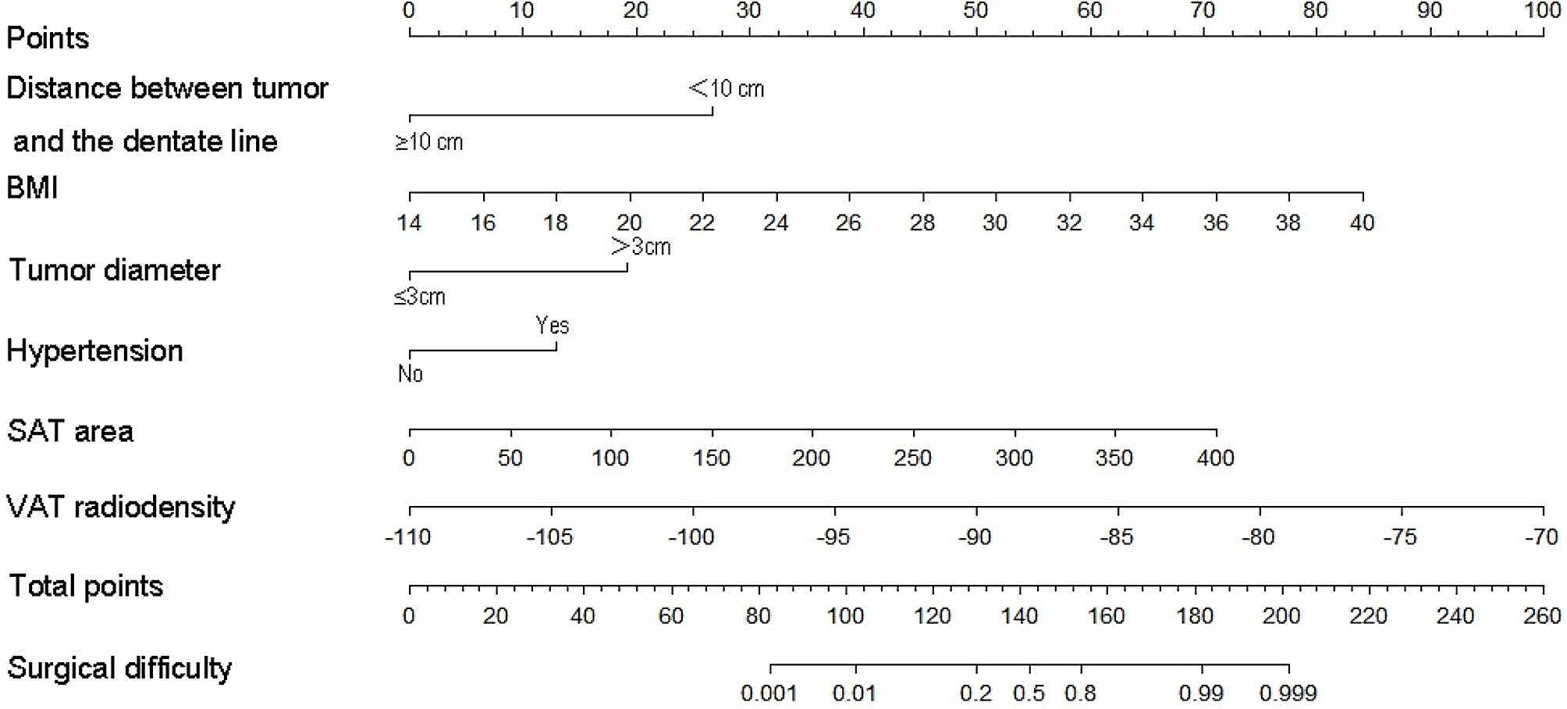
Predictive modeling of surgical difficulty nomograms

**Fig. 4:**
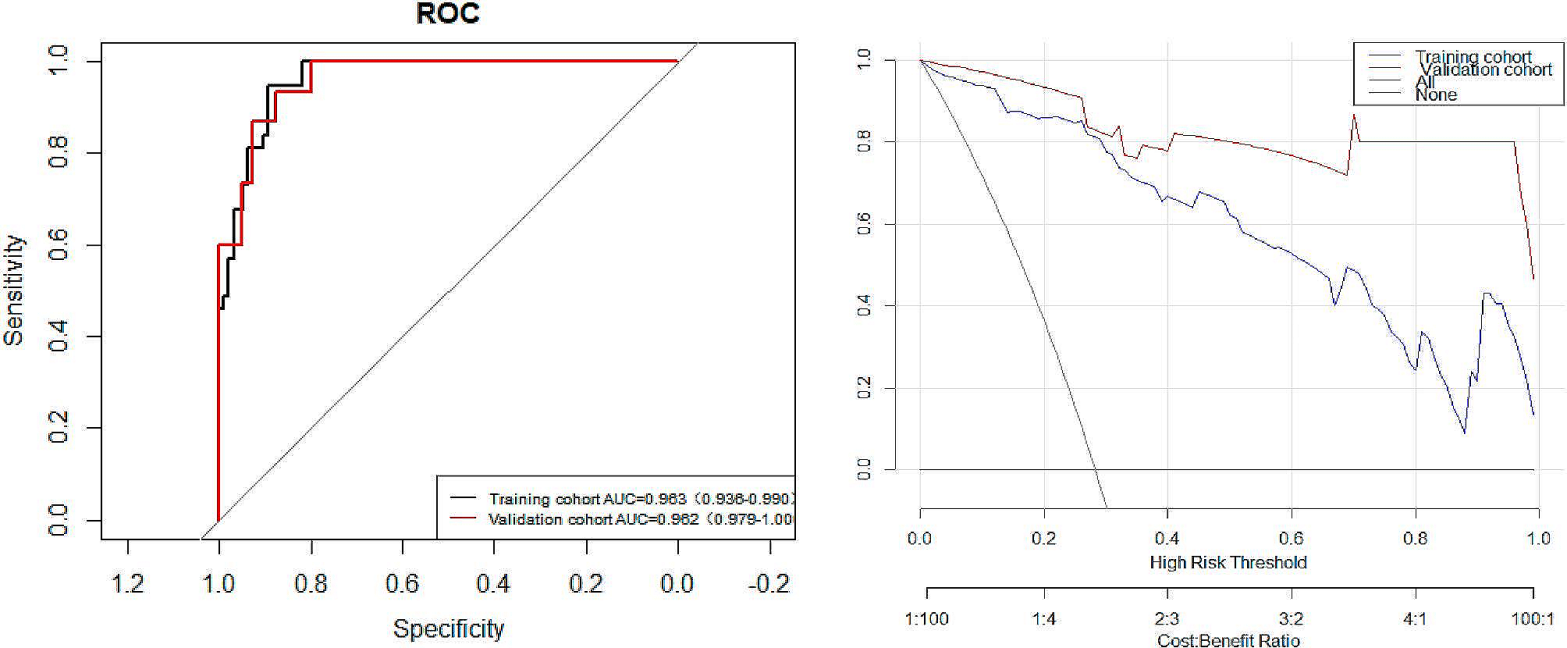
(**a**) Receiver Operating Characteristic curves (ROC) were utilized to predict surgical difficulty for both the training and validation cohorts. The training cohort is indicated by the solid black line, and the validation cohort is indicated by the solid red line. (**b**) The clinical utility is evaluated by performing a Decision Curve Analysis (DCA) analysis. The y-axis represents the net benefit, while the x-axis represents the threshold probability. The training cohort is indicated by the solid blue line, and the validation cohort is indicated by the solid red line

**Fig. 5:**
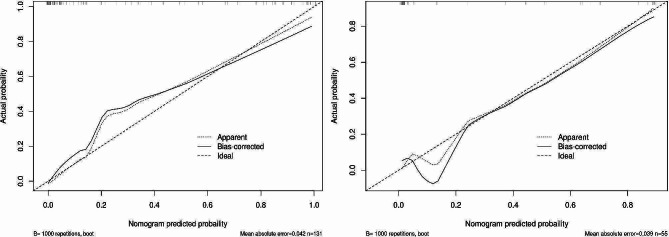
Calibration curves for the model provide a predictive risk assessment of surgical difficulty for both the training cohort (**a**) and the validation cohort (**b**). The solid line in the figure indicates the performance of the predictive model, with closer proximity to the diagonal dashed line indicating more accurate predictions. The calibration curves for the training cohort and validation cohort are in high agreement with the fitted line, indicating the high accuracy of the nomogram

To make the evaluation more intuitive, we created nomogram based on logistic regression, as logistic regression makes it easier to interpret nomogram. In the nomogram model, variable selection was based on a backward stepwise screening using the Akaike information criterion requiring *P* < 0.05: BMI, tumor distance from the dentate line, tumor diameter, VAT radiodensity, SAT area, and comorbid hypertension were included in the variables used to construct the nomogram(Fig. 3). The C- indices are all greater than 0.9, indicating that the predictive ability of the model has a high degree of confidence(Fig. 4a). Furthermore, the Decision Curve Analysis (DCA) showed significantly better net benefit in the predictive model(Fig. 4b). The calibration curves for this nomogram demonstrated favorable concordance (Bootstrap = 1000 repetitions, mean absolute error (training cohort) = 0.042, mean absolute error (validation cohort) = 0.039) (Fig. 5), and we also performed the Hosmer and Lemeshow tests, which indicated that both the training cohort and validation cohort indicated a good fit (training cohort *P* = 0.853; validation cohort *P* = 0.400).

## Discussion

In this study, four machine learning models were developed and validated for predicting the difficulty of LaTME. Based on the comparison of the ML models, all four ML models showed high performance, with the AUC of the SVM standing out in the training and validation groups, but not significantly different from the other models. Meanwhile, to further visualize the assessment of surgical difficulty, we also constructed a logistic regression-based nomogram, according to which clinical surgeons can use the nomogram to calculate the risk probability of surgical difficulty, to make adequate preoperative and intraoperative preparations.

LaTME is perhaps the most challenging type of surgery in colorectal surgery. Appropriate pelvic debridement and total mesorectal excision (TME) are essential to prevent local recurrence [20]. Previous studies have focused on the consequence of pelvic factors and rectal mesenteric fat area on the surgical outcome of lower and middle rectal cancer [21, 22], the consequence of a large vertical pelvic depth, a small pelvis, a short transverse meridian, a large sacrococcygeal curvature and a high rectal mesenteric fat area on the difficulty of the procedure was also determined [17, 23, 24], and these factors are particularly significant for men [25]. However, the relationship between quantitative pelvic measurements and surgical difficulty is uncertain, and some studies have even found no association between pelvic measurements and surgical difficulty [26]. Our study demonstrated that abdominal adipose tissue is an independent consequence of the difficulty of laparoscopic rectal cancer surgery. The assessment of abdominal visceral fat is important because it can result in the intraoperative separation of the visceral layer from the abdominal fascia, the exposure of the rectal vessels, and the smoke when applying the ultrasonic knife [27]. Therefore, we included VAT in the surgical difficulty assessment score to further refine the preoperative assessment of surgical difficulty. We found that there was a significant difference in the radiodensity of visceral adipose tissue between the two groups of patients, and according to one study, the radiodensity of fat was also associated with overall survival and mortality in colorectal cancer, and it was also demonstrated that this phenomenon may be due to: inflammation, browning of the adipose tissue, and edematous disorders [16, 28], and therefore, we hypothesized that the consequence of the radiodensity of fat on surgery may also be related to this, which, of course, requires further exploration and discussion.

BMI is considered to be the most common indicator describing overall obesity [29, 30], and previous studies have shown that high BMI has a significant consequence on postoperative outcomes after rectal surgery [4, 31, 32], but in recent years an “obesity paradox” has emerged [33–35]. A meta-study showed that obese patients (including class I/II/II) had a lower mortality rate within 30 days than patients with normal BMI, but a higher mortality rate after 30 days than patients with normal BMI [33], which reflects the limitations of BMI and the value of our study.

Our study also indicated that hypertensive patients are more difficult to operate, and a study has proved that the prevalence of hypertension is higher in patients with abdominal visceral obesity [36], therefore, hypertensive patients may still be due to abdominal visceral obesity, of course, we didn’t do more research and have no direct evidence to prove this, so I believe that we will have a report on this in the future.

Escal et al. [17]and other studies [37] included blood loss in the scoring criteria, and we considered that in some surgeries, which are interfered with by factors such as abdominal lavage, the measurement of blood loss may not be completely accurate, which may result in the grouping of surgical difficulty in some patients. Therefore, to formulate the prediction model more objectively, to make the model more persuasive, and to facilitate the generalization of the model, we did not include blood loss in the scoring factors, and we do not deny that intraoperative blood loss may reflect the difficulty of surgery to a certain extent. In the future, we will also include standardized measurements of blood loss in the scoring criteria.

In addition, robotic total rectal mesentery resection is now widely used, and several studies have shown that robotic total rectal mesentery resection is non-inferior to laparoscopic total rectal mesentery resection in terms of both short-term outcomes of postoperative complications and overall survival [25, 38–41], and even superior to laparoscopic total rectal mesentery resection. The rigidity of laparoscopic instruments and the limitation of operating space may affect specimen quality, and the robotic device may overcome the above disadvantages of laparoscopic instruments, resulting in better specimen quality and reduced local recurrence [42]. Interestingly, VFA with rectal mesenteric adipose tissue did not have significant clinical significance for the postoperative pathological safety of robotic total rectal mesentery resection [43, 44], which needs to be validated by a large sample from a multicentric population.

Laparoscopic and open surgery are still the dominant procedures for the treatment of rectal cancer [45], and therefore this study remains essential. In addition to the factors we investigated that consequence in the difficulty of rectal surgery, several studies have demonstrated that a history of previous abdominal surgery, preoperative radiotherapy, surgeon’s proficiency, preoperative patient’s nutritional status, and other factors can influence the difficulty of laparoscopic surgery [46, 47]. Although various factors consequence the difficulty of surgery, we believe that the scoring criteria demonstrated by Escal et al. [17] are more objective, and we believe that more factors will be included in the scoring of surgical difficulty in the future, thus further improving the objectivity and persuasiveness of the scoring, and also providing ideas for the evaluation of other surgeries.

Of course, this study has a few limitations. First, this is a retrospective study with a small number of enrolled patients, so selection bias cannot be completely ruled out and a larger sample size from more centers is needed for further validation. Second, laboratory tests, clinicopathologic features, and abdominal CT parameters were included in this study, but since CT only measures the mean area of two planes of the abdomen and does not measure the volume of abdominal fat, errors may arise as a result; finally, we only selected some of the clinical biochemical indexes, and factors that were not included may lead to residual confounders.

## Conclusions

This study developed four ML models for evaluating surgical difficulty, all of which indicated excellent efficacy, and to further visualize the evaluation, logistic regression-based nomograms are created. Both the training cohort and validation cohort confirmed the excellent performance of the models, providing clinicians with easy-to-use tools to help them make accurate surgical decisions. Of course, further validation through multi-center and large sample sizes is needed to ensure the prediction effect.

### Electronic supplementary material

Below is the link to the electronic supplementary material.

Supplementary Material 1

Supplementary Material 2

Supplementary Material 3

## Data Availability

No datasets were generated or analysed during the current study.

## Abbreviations

LaTME: laparoscopic total mesorectal excision
LR: logistic regression
SVM: support vector machine
RF: random forest decision tree
DT: decision tree
BMI: body mass index
RC: rectal cancer
CT: computerized tomography
ML: machine learning
ASA: American Society of Anesthesiologists
SAT: subcutaneous fatty tissue
VAT: visceral adipose tissue
VFA: /BSA visceral fat area/body surface area
SIG: Systemic Inflammatory Grade
HU: Hounsfield units
AUC: Area under the receiver operating characteristic curve
ROC: Receiver operating characteristic curve
CI: Confidence interval
