# Supplementary material for: Development and validation of machine learning models and nomograms for predicting the surgical difficulty of laparoscopic resection in rectal cancer
Source: World J Surg Oncol. 2024 Apr 25;22:111. doi: 10.1186/s12957-024-03389-3 (PMC11044303; doi:10.1186/s12957-024-03389-3)

**Supplementary Fig. S1** **Schematic diagram at the level of the L4/5 spine in the used patient using Slice-O-Matic software. Blue represents subcutaneous adipose tissue, yellow represents visceral adipose tissue, red represents skeletal muscle, and green represents intermuscular fat.**


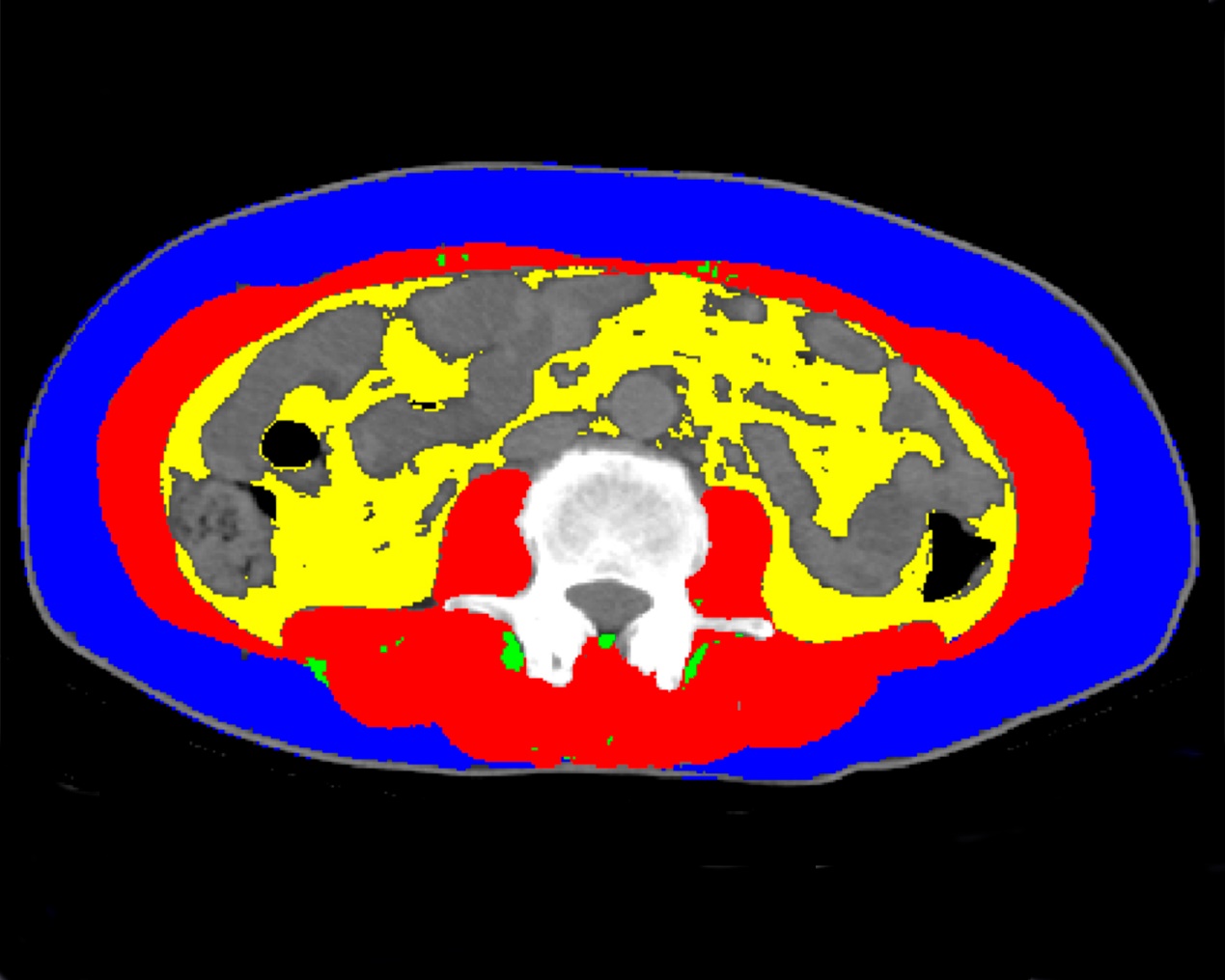

Supplement: Supplementary file 1 — Supplementary Material 1 [file 12957_2024_3389_MOESM1_ESM.docx]
